# Parametric Study of Jet/Droplet Formation Process during LIFT Printing of Living Cell-Laden Bioink

**DOI:** 10.3390/mi12111408

**Published:** 2021-11-16

**Authors:** Christina Kryou, Ioannis Theodorakos, Panagiotis Karakaidos, Apostolos Klinakis, Antonios Hatziapostolou, Ioanna Zergioti

**Affiliations:** 1 Department of Physics, National Technical University of Athens, 15780 Zografou, Greece; chkryou@central.ntua.gr (C.K.); jtheod@mail.ntua.gr (I.T.); 2 Biomedical Research Foundation Academy of Athens, 11527 Athens, Greece; pkarak@bioacademy.gr (P.K.); aklinakis@bioacademy.gr (A.K.); 3 Department of Naval Architecture, School of Engineering, University of West Attica, 12243 Athens, Greece; ahatzi@uniwa.gr; 4 Institute of Communication and Computer Systems, 15780 Zografou, Greece

**Keywords:** laser-induced forward transfer, time-resolved imaging, laser fluence, cell-laden bioink, distance, droplet/jet impingement

## Abstract

Bioprinting offers great potential for the fabrication of three-dimensional living tissues by the precise layer-by-layer printing of biological materials, including living cells and cell-laden hydrogels. The laser-induced forward transfer (LIFT) of cell-laden bioinks is one of the most promising laser-printing technologies enabling biofabrication. However, for it to be a viable bioprinting technology, bioink printability must be carefully examined. In this study, we used a time-resolved imaging system to study the cell-laden bioink droplet formation process in terms of the droplet size, velocity, and traveling distance. For this purpose, the bioinks were prepared using breast cancer cells with different cell concentrations to evaluate the effect of the cell concentration on the droplet formation process and the survival of the cells after printing. These bioinks were compared with cell-free bioinks under the same printing conditions to understand the effect of the particle physical properties on the droplet formation procedure. The morphology of the printed droplets indicated that it is possible to print uniform droplets for a wide range of cell concentrations. Overall, it is concluded that the laser fluence and the distance of the donor–receiver substrates play an important role in the printing impingement type; consequently, a careful adjustment of these parameters can lead to high-quality printing.

## 1. Introduction

Currently, various bioprinting technologies are emerging as potential methods for the multidisciplinary field of tissue engineering and regenerative medicine [1,2,3]. The main goal of bioprinting is the positioning of multiple cell types on a supporting substrate in a precise manner in order to recapitulate the complex cell–cell and cell–environment interactions. The post printing cell viability and the spatial resolution are key parameters for the overall efficacy of the printing process. Different bioprinting techniques can be employed, including drop-on-demand techniques, such as inkjet printing [4], laser-based printing [5,6], as well as extrusion printing [7]. Among all these technologies, laser-induced forward transfer (LIFT) is a laser-based printing technique that enables the simultaneous transfer and patterning of material from a donor to a receiver substrate, with a lateral resolution down to a few micrometers [8]. As described in the literature, this technique has been used in various solid or liquid printing applications, including organic biomaterials [9,10,11], metals [12,13], and biological compounds [14,15,16,17]. The LIFT technique has been applied to print with various high-precision biomaterials, including proteins [18], DNA [19], living cells [20,21,22,23], and cell-encapsulating hydrogels [24,25]. In the case of liquid LIFT, the transfer is initiated in the form of a liquid jet that draws liquid from the donor film onto the receiver substrate. The traditional LIFT technique relies on the absorption of the laser pulse directly into the material under transfer. However, in the case of carrying out the LIFT with biological materials sensitive to the laser radiation, the donor substrate is pre-coated with an absorbing layer (a few nanometers), usually a metallic or polymeric layer, to protect those materials. An alternative method to LIFT, with a much thicker absorbing polymer layer (a few micrometers), is blister-actuated LIFT (BA-LIFT). Upon ablation of the polymer film, the expansion of the trapped gasses results in a blister on the polymer layer that converts the laser and the chemical energy released during laser ablation, into a mechanical impulse that propels the material under transfer towards the receiving substrate [26]. Compared with other bioprinting techniques, such as orifice-based inkjet printing, which has certain limitations, such as nozzle clogging [4,27], LIFT, as an orifice-free printing approach, has advantages in direct writing viscous materials (1–300 mPa/s) [28]. Moreover, LIFT achieves high-resolution printing at cell concentrations up to 1 × 10^8^ cells/mL [7]. The setup for LIFT printing consists of a pulsed laser source and two positioning systems: a transparent donor substrate coated with a thin energy-absorbing layer (also called the dynamic release layer (DRL)) carrying the material under transfer (liquid or solid form), and a receiver substrate, which is placed opposite and parallel to the donor surface. The distance between the donor and receiver surfaces is usually not a limiting parameter; it can range from a few tens of micrometers up to a few millimeters. In brief, laser pulses are focused into the thin energy-absorbing layer, which is vaporized locally in the focal region of the laser beam. As a result of the laser absorption, a high-pressure bubble forms inside the liquid and it rapidly expands to produce a fast and thin jet, with the subsequent separation of one or more droplets, and its transfer to the receiver substrate. Nevertheless, the mechanism which leads to droplet ejection and deposition is still not well understood. Two different hypotheses have been proposed. According to the first one, printing relies upon the ejection of a single droplet that reaches the substrate [29], whereas the second one claims that printing is achieved through the formation of a long jet that contacts the receiver substrate [30]. In order to elucidate this, time-resolved imaging of the LIFT of liquids greatly helps to understand the transfer dynamics in order to optimize the printing process. In addition, the experimental parameters associated with LIFT are related to the pulse energy and the focusing conditions [25], the substrate composition [29], and the donor–receiver substrate distance [18,31]. During LIFT printing, three jetting regimes are usually described: the subthreshold, well-defined jetting, and plume regimes. It has also been demonstrated that the cell-printing deposition and the cell viability depend on jet dynamics and the jet impact with the receiver substrate, which are controlled by the rheological properties (viscosity, density, and surface tension) [32,33], and by the distance between the donor and the receiving substrate [34,35,36].

The present study focuses on assessing the effect of the cell concentration on the transfer mechanism in the case of Newtonian liquids, and its correlation with the morphology of the printed droplets. The study explores the jetting dynamics and the resulting printing quality during the LIFT of bioinks, with different cell-concentration bioinks. There are two main successive events during LIFT printing: jet/droplet formation, and jet/droplet impingement and deposition. To fully understand the potential of LIFT printing, it is necessary to study both the jet/droplet formation and the deposition dynamics. The process conditions for LIFT printing, including the laser fluence and the distance between the donor–receiver substrate, have been discussed in detail.

## 2. Materials and Methods

### 2.1. Experimental Setup

**Figure 1:**
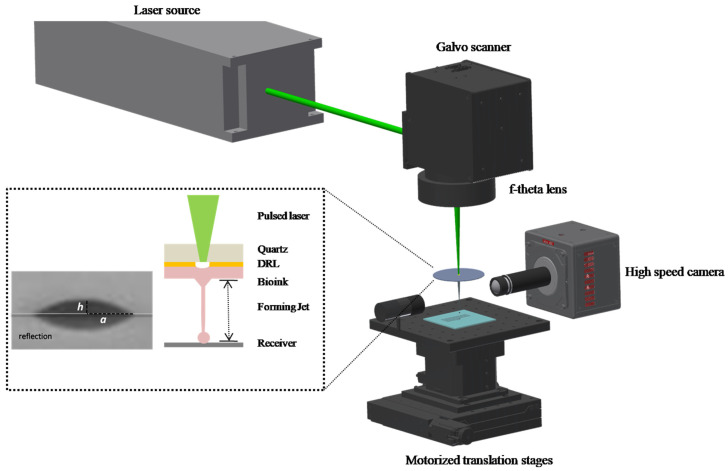
Schematic of LIFT setup.

The experimental setup used for these experiments consisted of two main subsystems: the LIFT printing setup and a high-speed imaging system, as depicted in Figure 1. All experiments were performed using a single-laser pulse process, per printed-droplet or multiple-droplet printing, in line configuration. The LIFT printing setup consisted of a nanosecond DPSS Nd: YAG laser (NANIO-532-20-V-100, InnoLas Photonics GmbH, Krailling, Germany), operating at a wavelength of 532 nm, with 20 W maximum output power, and a 500 kHz maximum repetition rate. In this work, repetition rate values from 1 to 10 kHz were used, where the pulse duration was about 20 ns. The laser beam energy was tuned with a half-wave plate/polarizer attenuation setup, while a two-lens telescopic setup was used to transform the beam’s size into the desired 14 mm input size for the galvanometric scanning head. The latter was an intelliSCAN-III-14 (SCANLAB GmbH, Puchheim, Germany) with a maximum scanning speed at 5 m/s, combined with an f-Theta lens with a focal length of 170 mm, through which the laser beam was focused on a spot size of about 60 μm beam waste at the working distance of the scanning head, where the donor substrates were placed.

The donor substrates were made of glass slides (26 × 76) mm^2^ DELTALAB, Barcelona, Spain) and they were coated with a thin laser-absorbing layer of gold (60 nm) using a sputter coater. The receiver substrate comprised either a sterilized glass slide substrate for the high-speed cell printing visualization experiments, or a sterilized glass slide substrate of a 13 mm diameter (CCVN-013-100, Labbox, Milan, Italy), coated with gelatin for the cell-printing experiments. The different receiver substrates were positioned onto a motorized three-axis translation stage, and the distance between the donor and receiver substrates was varied from 500 to 2000 μm. The LIFT printing experiments were conducted under different conditions. In particular, the first experiments were performed using a repetition rate of 1 kHz to investigate the jet morphology of the cell-free and cell-laden bioinks, as well as the printed droplet diameter, the jet velocity, and the printed volume at different laser fluences, ranging from 280 to 900 mJ/cm^2^. The minimum value is the lowest workable laser fluence to produce droplet ejection (threshold), and the maximum is the highest value employed in our experimental conditions. The second set of experiments was performed to evaluate the effect of the donor–receiver distance during the LIFT printing of cell-laden bioinks at 500 mJ/cm^2^. This laser fluence was selected since the high-speed experimental results indicated that, at this fluence, a stable and reproducible jet is produced.

The high-speed imaging setup consisted of a high-speed camera (Mini AX-100, Photron USA, Inc., San Diego, CA, USA) coupled to the system (Figure 1), with a maximum recording speed at 540 kfps, and a standard LED (LEDD1B, Thorlabs GmbH, Lübeck, Germany) placed opposite of the camera for illumination purposes. In this work, a recording speed at 127.5 kfps, equivalent to a time resolution of 7.8 µs, was used for monitoring the emerging jet. The LED was focused on the liquid jet’s formation plane, perpendicular to the donor–receiver substrates. The synchronization of the triggering of the laser camera was achieved by custom LabView software. A simple two-lens arrangement, with a 1.5× optical magnification, was used to adjust the donor–receiver distance at the camera sensor size.

### 2.2. Cell Culture

The human breast cancer cell line, MDA-MB-468, was purchased from ATCC (LCG standards, Teddington, UK) and cultured in Dulbecco’s modified Eagle’s medium (DMEM, high glucose, SH30243.01, Cytiva, Marlborough, USA), supplemented with 10% fetal bovine serum (FBS, FBS12A, Capricorn Scientific, Ebsdorfergrund, Germany), and 1% penicillin/streptomycin (15140-122, Gibco, Thermo Fisher Scientific, Waltham, MA, USA), in a humidified atmosphere of 5% CO_2_ at 37 °C. The MDA-MB-468 routinely passaged every 2 or 3 days and tested for mycoplasma.

To prepare the bioinks for printing, exponentially growing MDA-MB-468 were trypsinized (T4049, Sigma-Aldrich, St. Louis, MO, USA), and the viable ones were counted on a hemocytometer via trypan blue (15250-061, Gibco) exclusion. The cells were then centrifuged at 1500 rpm for 5 min, and resuspended at 2.5, 7.5, and 20 × 10^4^ cells/μL, in medium with or without 10% glycerol (autoclaved sterile, 40058-ATO, Lach-Ners.r.o.). The resuspended cells were kept on ice until direct printing, normally within one hour.

After printing, the cells (coverslips) were transferred into 24-well plates, and 0.5 mL of complete medium was added to each well.

### 2.3. Cell Viability Assay

The printed cells were incubated with Hoechst 33,258 (10 μg/mL) and propidium iodide (PI, 1 μg/mL, 40017, Biotium) in the culture medium for 15 min. Hoechst 33,258 is cell-permeable dye, while PI is not, enabling the discrimination between live and dead cells. After the incubation, the cells were washed 3 times with PBS and immediately visualized.

### 2.4. Preparation of Donor Substrate

For all the presented printing experiments, 1-mm-thick glass slides, (26 × 76) mm^2^, were cleaned by sequential exposure to isopropyl alcohol, deionized water, and acetone in an ultrasonic treatment for 30 s, followed by purging with purified air, and then coating with a thin gold film of a 60-nm thickness. A volume of 3 μL of the cell suspension was applied in order to obtain an approximately 90µm-thick coating (see Section 2.5).

### 2.5. Preparation of Receiver Substrate

For the cell-printing experiments, sterilized and gelatin-coated receiving glass coverslips (13 mm in diameter, CCVN-013-100, Labbox, Milan, Italy) were utilized. The gelatin was used as a cell adherent receiver substrate. In brief, autoclave sterilized coverslips were submerged in ultrapure water, with 0.1% gelatin (ES-006-B, Millipore, Burlington, VT, USA), for at least 30 min in a laminar flow hood. Then, the coverslips were air-dried in the hood and used for bioprinting within the day.

### 2.6. Bioink Composition

DMEM (Bioink A) is widely used in biology for supporting the growth of many different mammalian cells [37], and it was used as a first choice of the cell-free bioink printing. The second cell-free bioink (Bioink B, DMEM +10% glycerol or (DMEM-G)was selected in order to study the effect of the additives. Bioink B consisted of a model solution designed to create a stable liquid layer over the duration of the experiment. The viscosity was slightly increased, mainly using glycerol. Moreover, glycerol was added for its wide biomedical applications and its low vapor-pressure limit evaporation, and for the potential drying of printed biomaterial [38,39]. Cell-laden bioinks in this study were prepared by resuspending the MDA-MB-468 cell line into DMEM or DMEM-G (+10% glycerol) to make different cell-laden bioinks, with concentrations of 2.5, 7.5, and 20 × 10^4^ cells/μL. The material properties of the cell-free bioinks are shown in Table 1. The dynamic viscosity, as well as the density, of the bioinks, A and B, were evaluated at 20 °C, using a stress-imposed rheometer. We decided not to measure the viscosity of the different cell-laden bioinks (Bioinks C–G, Table 1) because the high stresses generated during the rheometer experiments could lead to large inhomogeneities within the bioinks [40].

### 2.7. Measuring Droplet Diameters

The droplet diameters were measured based on the average value of the four representative printed droplets for each case after their equilibrium on the receiver substrate. For those droplets followed by secondary droplets, their equivalent feature diameter was estimated based on their equivalent total volume, calculated by assuming a spherical cap segment of all the droplets, using the following, Equation (1):

(1) $$V_{\mathrm{cap}}\;=\;\frac{1}{6}\pi h\;(3a^{2}\;+\;h^{2})$$

where h and a represent the height of the jet and the base radius, respectively.

### 2.8. Imaging and Analysis

Brightfield or fluorescent images were obtained on a Leica DMIRE2 microscope through an ORCA-flash 4.0 LT+ digital camera (model C11440, Hamamatsu Photonics Deutschland GmbH, Herrsching am Ammersee, Germany). Hoechst 33,258 and PI were visualized through A4 and N21 filter cubes, respectively. ImageJ software was utilized to process the captured images.

### 2.9. Statistical Analysis

The statistical analysis of the data value distribution was performed by calculating the mean and its standard deviation (STDEV) from four samples/cases, with regard to the diameter and volume of the printed droplets, and, for the survival, at least 9 individual droplets per condition were measured.

## 3. Results and Discussion

Ultimately, the purpose of this study was to examine the suitability of the LIFT technique for the controlled transfer of cell-free and cell-laden bioinks, which is a major technological challenge for most 3D bioprinting techniques. Specifically, this article presents the laser printing of cell-laden bioinks with three different cell concentrations and highlights the importance of the laser fluence and the distance between the donor and receiver substrates for the laser printing outcome.

### 3.1. Ejection Mechanism

In order to understand the reported dependence of the jet morphology on the laser fluence, and to correlate it with the liquid ejection mechanism, time-resolved imaging was carried out at three different laser fluences for all bioinks examined. We analyzed the jetting regime to study the effect of the laser energy on the jet velocity and jet morphology. The time-resolved images of jets from different laser fluences, ranging from 280 to 900 mJ/cm^2^, were captured. In all images, the laser beam is impinging the donor substrate from above, with the receiver substrate being placed at a distance of 500 μm with respect to the donor substrate.

**Figure 2:**
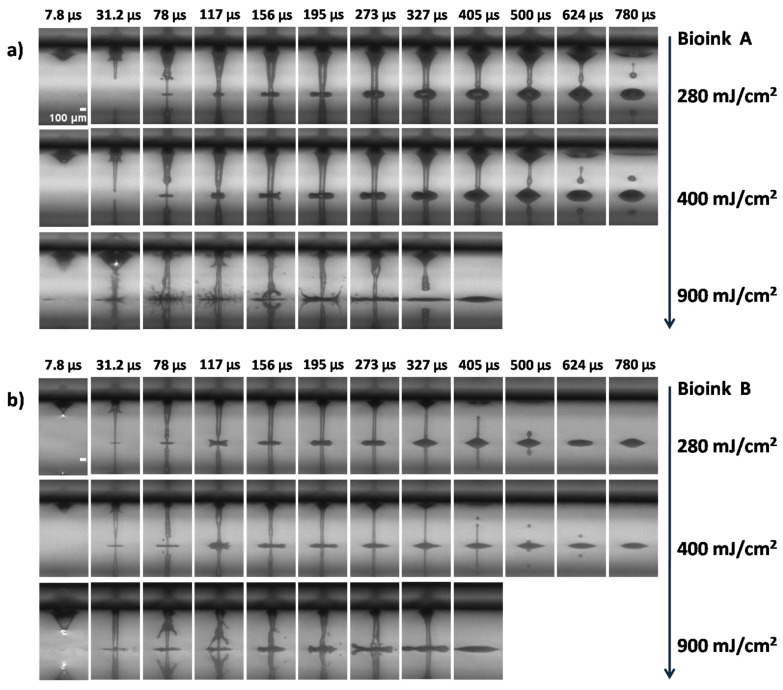
Time-resolved images of the LIFT printed cell-free bioinks at different laser fluences: (**a**) Bioink A; (**b**) Bioink B. In every image, the laser beam is impinging the donor substrate from above, with the receiver substrate being placed at a distance of 500 μm with respect to the donor substrate. Scale bar: 100 μm.

**Figure 3:**
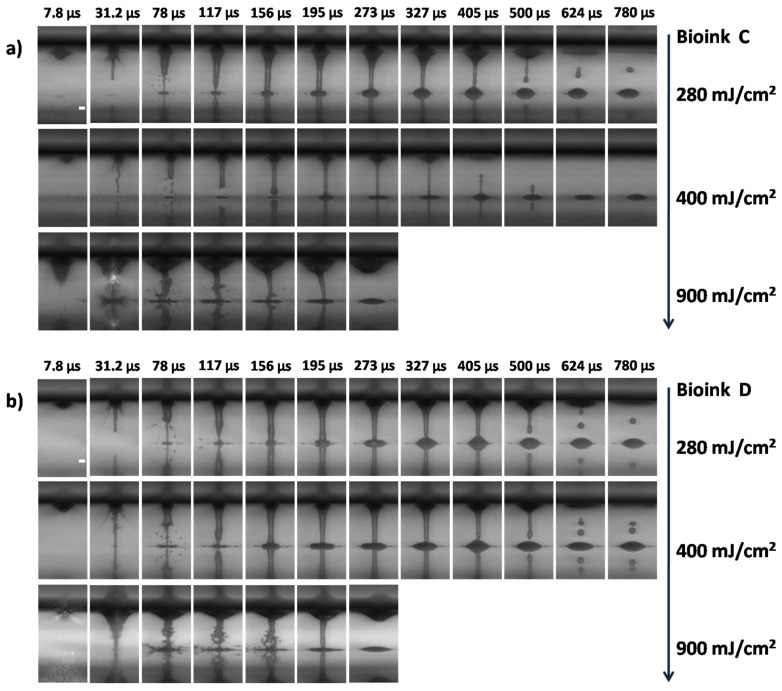

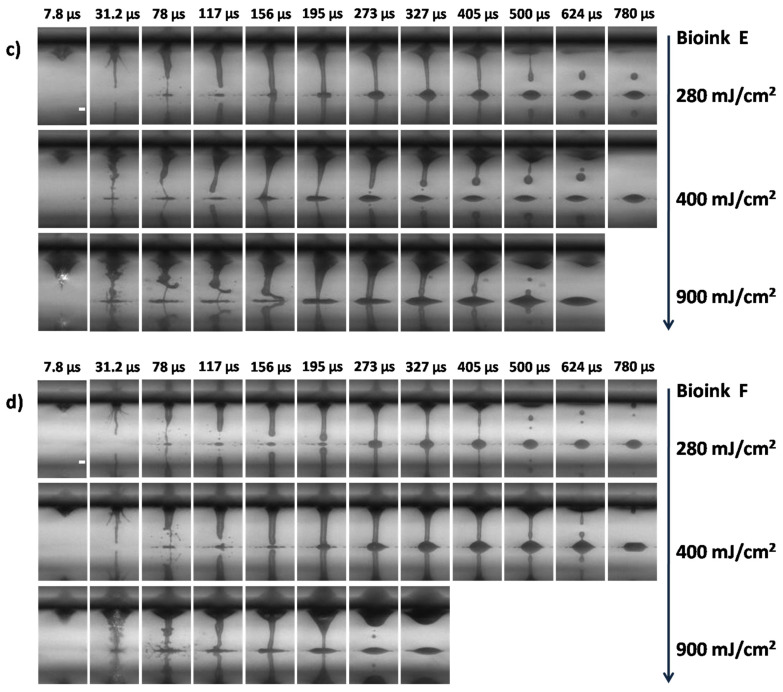

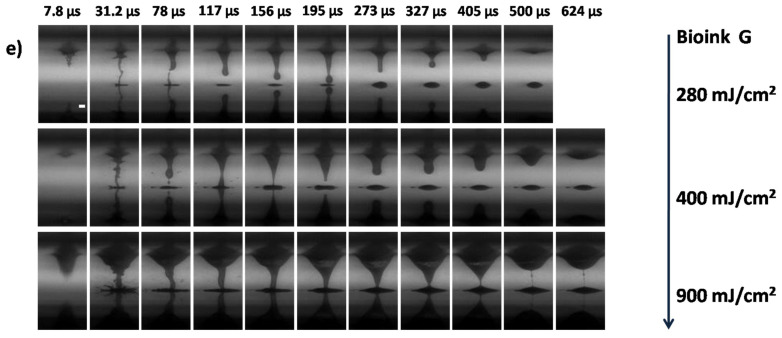
Time-resolved images of the LIFT-printed cell-laden bioinks at different laser fluences. (**a**) Bioink C; (**b**) Bioink D; (**c**) Bioink E; (**d**) Bioink F; (**e**) Bioink G. In every image, the laser beam is impinging the donor substrate from above, with the receiver substrate being placed at a distance of 500 μm with respect to the donor substrate. Scale bar: 100 μm.

The process of LIFT printing is triggered by the absorption of the laser pulse energy at the interface between the DRL and the material under transfer on the donor holder. Figure 2 and Figure 3; show a series of time-resolved images of cell-free and cell-laden bioink ejection and deposition on the receiver substrate. All images acquired from videos recorded (at 127.5 kfps) using a high-speed camera.

A sequence of images from the ejection of cell- free bioinks (Bioinks A, B) is presented in Figure 2a,b and the ejection of cell-laden bioinks in Figure 3a–e (Bioinks C, G). As a consequence of the laser energy absorption, a vapor bubble is created in the material under transfer. The gas pressure produces the expansion of the bubble, pushing the material forward. This expansion creates high pressure in the material under transfer around the bubble border, which promotes the flow of the liquid material along the bubble wall and towards its pole, giving place to the development of a thin needle-like jet [41]. The cavitation-bubble collapse is followed by the progression of the jet front that propels the material under transfer towards the receiving substrate, usually at speeds ranging between tens and hundreds of meters per second, depending on the laser fluence [42]. Despite the high jet velocity, the formation of long thin stable jets leads to the gentle deposition of the droplets without satellites [30,42]. Shortly after the arrival of the jet on the receiver substrate, the liquid, which is in contact with the solid surface, undergoes a spreading process. Initially, the landed droplet takes the form of a flattened sphere that expands while maintaining its shape, until further liquid is ejected from the drop base, continuing the spreading process.

Actually, the initial flat shape of the printed droplet corresponds to the first 78 μs, as depicted in Figure 2a,b and Figure 3a–e (similar jet evolution, as a function of laser fluence, for all bioinks). At this point, after a few microseconds, the jet continues to feed the growing droplet, resulting in an increase of the printed volume and the contact angle of the droplet until the jet breaks up because of Plateau–Rayleigh instability. This breakup is driven by the liquid surface tension, which aims to reduce the surface energy of a system. As long as perturbations are growing along the jet neck, they will cause the jet to break into drops, a state where the surface energy is well below that of the full jet [43,44].

The viscosity of the bioinks is of primary importance in the hydrodynamics of the jet resulting in printing [45]. At low pulse energies, the viscosity might play a more important role in the jet behavior [46]. Thus, the similar jet evolutions, as a function of the laser fluence in Figure 2a,b for the cell-free bioinks (Bioinks A and B), might be explained by their close viscosities (2.1755 mPa.s for Bioink A, and 2.3767 mPa.s for Bioink B). For low viscosity bioinks, such as those employed in the present study, jetting develops at low laser fluences (at 280 mJ/cm^2^ and 400 mJ/cm^2^, Figure 2a,b and Figure 3a–d). A visual analysis of the LIFT through high-speed imaging demonstrated that the formation of stable jets during distancing from the donor is followed by well-defined bioink droplets. As the jet progresses and impinges on the receiver substrate, it begins to accumulate liquid at the impact site, with practically no deviation from its straight trajectory, maintaining its well-defined shape. From the time-resolved images of Figure 3a–d, it is shown that bioinks with glycerol (Figure 3b,d) produced a more stable jetting behavior during printing and, for this reason, they were selected for further experiments that studied the effect of the laser printing distance on the printed droplet diameter and volume. Therefore, the printing results with bioinks without glycerol that we chose to include are only at the optimum laser fluence of 500mJ/cm^2^. In order to determine how the effect of the cell concentration affects the bioink jet behavior, which is useful for the printing behavior, we studied the influence of such a high bioink concentration (Bioink G, in Figure 3e). This bioink is quite unstable; as a result, we have tested it only to confirm the trend.

However, for higher laser fluences (e.g., 900 mJ/cm^2^ in Figure 2 and Figure 3), the produced jets become unstable and non directional, while their flow can be characterized as turbulent. This jetting behavior is observed when the pressure of the gas that is entrapped within the cavitation bubble exceeds the value of the outside pressure and facilitates the evolution of the jet by overcoming the cohesive forces of the surrounding liquid film, leading to the violent propulsion of both liquid and gas [47]. On the contrary, for laser fluences just below the ejection threshold (~200 mJ/cm^2^), jets can also be formed; however, they do not evolve towards the receiver substrate because the jet velocity is insufficient. Instead, they recoil with bubble collapse [46,47]. Finally, our preliminary viability assays indicated that the cell survival rate and the printing accuracy deteriorate for laser fluences above 900 mJ/cm^2^.

From the time-resolved images of the cell-free and cell-laden bioinks, it is clear that stable liquid ejection occurs only at lower laser fluences, and that the transition between the stable and unstable jets is quite abrupt. In other words, regular droplets are obtained at lower fluences, irregular features and satellites become present at the transition fluence of 900 mJ/cm^2^, and splashes/satellites are evident at the highest fluence, corresponding to liquid ejection with bursting.

### 3.2. Jet Formation and Breakup during LIFT Printing

**Figure 4:**
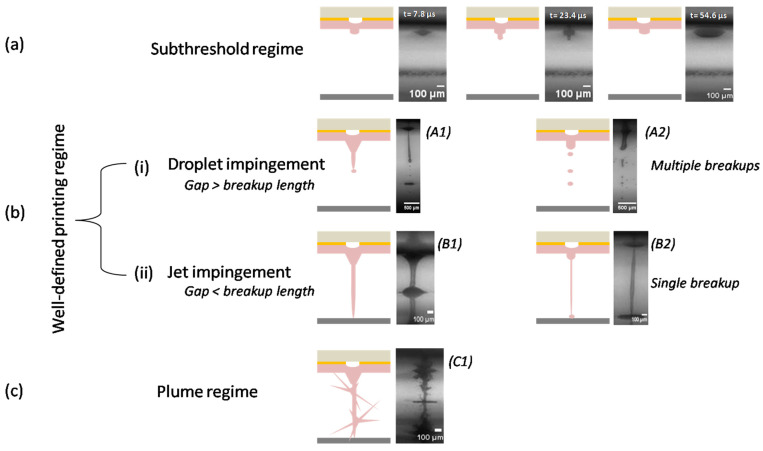
Schematic of jet formation (and respective experimental results of Bioink F) as a function of different distances between the donor–receiver substrate: (**a**) Subthreshold regime, morphological feature of a forming jet from 7.8–54.6 μs, during printing of Bioink F, under 200 mJ/cm^2^ and a 500 μm distance between the donor–receiver substrate; (**b**) Well-defined printing regime where: (i) Droplet impingement; (A1) morphological feature of a forming jet at 327 μs during printing of Bioink F at 500 mJ/cm^2^ and a 1500 μm distance between the donor–receiver substrate; and (A2) morphological feature of a forming jet at 273 μsduring printing of Bioink F at 500 mJ/cm^2^, with a 2000 μm distance between the donor–receiver substrate; (**c**) Plume regime; (C1) morphological feature of a forming jet at 46.8μs during printing of Bioink F at 900 mJ/cm^2^ and a 500 μm distance between the donor–receiver substrate.

The jetting morphology and breakup mechanism during LIFT printing was investigated using time-resolved imaging [48,49]. As indicated from this study, there are three distinct jetting behaviors during the laser printing of the cell-free and cell-laden bioinks. Specifically, they can be classified into: the subthreshold, well-defined jetting, and plume regimes, as depicted in Figure 4. In the subthreshold regime, the forming jet returns back to the donor substrate without transferring material. In the plume regime, the breakup of the initial jet and the jet-forming spray led to the formation of undesirable droplets dispersed over a large area of the receiver substrate. As a result, both of these regimes are considered undesirable during LIFT printing. During a well-defined printing, with/without the initial bulgy shape, a forming jet could break up before impinging the receiver substrate. This condition depends on whether the distance between donor–receiver substrates is greater than the breakup length or not. As a consequence, two different cases show up: (i) droplet-impingement printing, in which the distance between the donor–receiver substrates is greater than the breakup length. A well-defined jet could break up into several droplets before landing onto the receiver substrate; and (ii) jet-impingement printing, in which the distance between the donor–receiver substrates is smaller than the breakup length. A well-formed jet reached the receiver substrate before its breakup (either single or multiple breakups). Throughout droplet- and jet-impingement printing with multiple breakups, the trajectory of the droplets is affected by random perturbations. Specifically, it is observed that, during droplet-impingement printing, a jet breaks up into several non directional droplets and deposits onto the receiver substrate, resulting in several fragments being dispersed around the primary printed droplet. For the jet-impingement printing condition with multiple breakups, the jet keeps thinning and accumulating on the first printed droplet onto the receiver substrate. However, in low-viscosity materials, the thinned ligament breaks up into a few fragments, resulting in secondary droplets dispersed over the first printed droplet and, as a result, the printing quality decreases. The presence of the secondary droplets during LIFT printing does not seriously affect the quality of the final droplet, as long as the majority of the fragments merge with the first printed droplet.

### 3.3. Effect of Cell-Laden Bioink (and Cell-Free Bioink) Concentration on Printed Droplet Size

**Figure 5:**
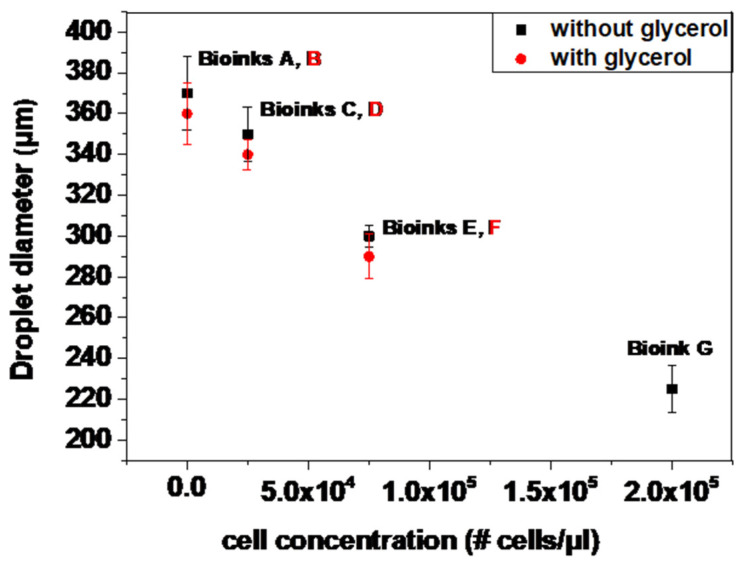
Droplet diameter as a function of cell concentration at 500 mJ/cm^2^. Error bars depict STDEV, *n* = 4 droplets per condition.

The effect of the cell concentration on laser printing, in terms of the printed droplet size, was also investigated during the LIFT printing of different cell-laden and cell-free bioinks, at a constant laser fluence, and distance between the donor–receiver substrates (500 mJ/cm^2^ and 500 μm distances). For all bioinks, the printing condition was jet-impingement printing with a single breakup. As demonstrated in Figure 5, the droplet diameter decreases with the increase in the cell concentration (Bioink C–Bioink G). At a given laser fluence, this linear decrease should be attributed to the combined contributions of the cell density, surface tension, and viscosity [21]. The droplet diameter reduction is higher for high-cell-concentration bioinks [32]. This is probably related to the more incident energy needed for the system to overcome the viscous effects of higher-cell-concentration bioinks, rather than transferring a larger volume. Although the cell concentration of Bioink G is considered too high for laser bioprinting because the large number of cells with respect to the available medium will lead to dehydration and cell death, we decided to test this condition purely for the assessment of the effect of the cell concentration on the printing behavior, and not for its biological effects on the printed cells.

According to the literature, viscosity increases with the increase in the cell concentration at a given shear rate [50,51]. In general, these phenomena can be explained because most of the input laser energy is converted to the elastic, surface, and kinetic energies of the forming jets. As a result, the viscous dissipation during the droplet deposition increases and the remaining kinetic energy of a jet decreases, resulting in a smaller jet volume ejected and a lower droplet size and velocity [52,53,54].

The jet velocity was calculated based on the spatial position difference of the jet front of the two sequential imaging frames of the jetting process from the following, Equation (2):

V = (h_2_ − h_1_)/(t_2_ − t_1_), (2)

where h and t represent the height of the jet and time, respectively. Time-resolved imaging experiments using donor liquid films, with the thickness of around 90 μm, revealed the appearance of a thicker second jet following the first needle-like jet, which originates after a delay of several microseconds, and gradually thins its central part until it pinches-off.

**Figure 6:**
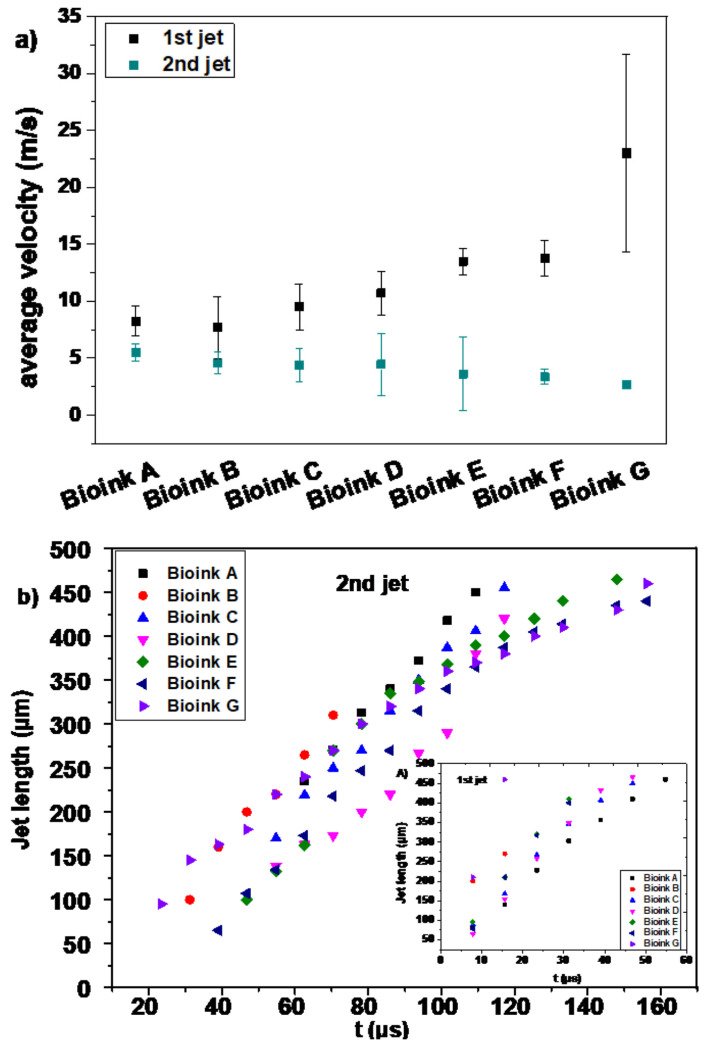
(**a**) Average jet velocities of all bioinks. Error bars depict STDEV, *n*= 4 droplets per condition; (**b**) Second jet length vs. time for all bioinks at 500 mJ/cm^2^. Bottom inlet, first jet length vs. time for all bioinks at 500 mJ/cm^2^.

Specifically, the analysis of the jet length versus time reveals that two different jetting regimes can be observed, each one presenting a linear relationship between position and time, at a constant laser fluence (500 mJ/cm^2^) (Figure 6b). The first regime (Figure 6b inset graph) represents the initial expansion stage (first jet), and the average velocities range from 8 to 22 m/s (Figure 6a), while the printed volume was estimated to be ~1/10 of the final printed volume. The second regime (Figure 6b) represents the jet formation and its propulsion process until its relaxation behavior begins, with the front velocity being about 4 m/s, i.e., 2.5 times lowers than that of the first jet. While Bioink G is the most concentrated bioink, the jet front velocity of the first jet was found to be higher than that of the four other less concentrated bioinks (and the cell-free bioinks). This can be correlated to the dramatic change in the viscosity of the bioinks, which could be attributed to its non-Newtonian behavior and the high shear rates generated during the experiment; the latter needs further investigation.

On the basis of the above, it can be concluded that the velocity of the second jet is the main factor that supports the efficient immobilization of the printed cells.

**Figure 7:**
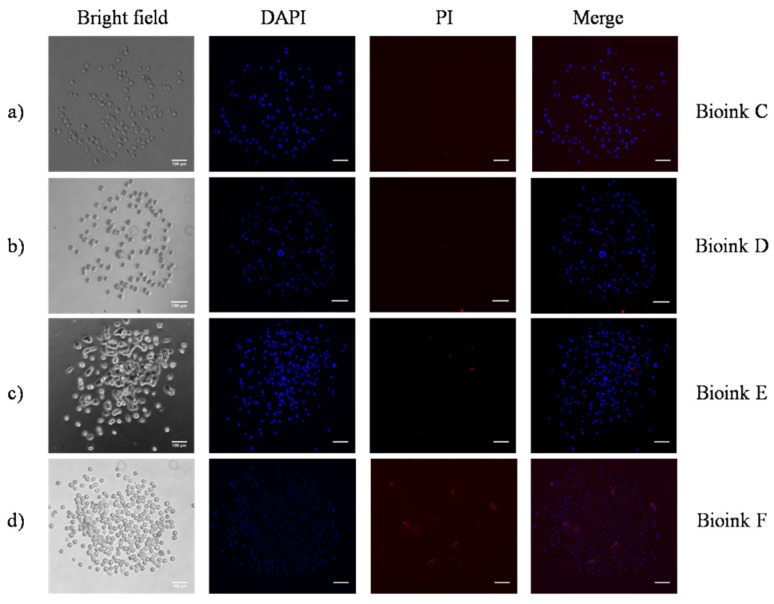
Microscopy images of the printed cell-laden bioinks at 500 mJ/cm^2^: (**a**) Bioink C; (**b**) Bioink D; (**c**) Bioink E; and (**d**) Bioink F, on the gelatin-coated glass slide 15 min after printing. To determine the cellular survival after printing, cells were stained with Hoechst 33,258 and PI (red PI staining indicates dead cells, while all printed cells are displayed in blue). Scale bar: 100 μm.

To determine the cell survival after printing, the process was performed with the same laser parameters for Bioinks C–F (500 mJ/cm^2^ laser fluence, and a 500 μm distance between the donor–receiver substrate), and the cells were stained with Hoechst 33,258 and PI. In this case, all the jets evolved laminarly towards the receiver substrate without splashing. The cell-laden bioinks (Bioinks C–F) were laser-printed to the gelatin-coated receiver surface, as shown in Figure 7. The nuclei of the printed cells appear in blue, while the dead cells appear in red. Table 2 shows the total number of printed and of dead cells 15 min after the transfer. For Bioinks C and D, such a result corresponds to an average cell survival after printing of 92%, and for the higher-cell-concentrated bioinks (Bioinks E and F), the average survival rate was 94%.

For the statistical analysis, the cell survival after transfer was also determined for laser fluences of 400 mJ/cm^2^ and 900 mJ/cm^2^, and a 500 μm distance between the donor–receiver substrate, as shown in Table 3. In the case of 400 mJ/cm^2^, the cell survival of Bioinks C and D was 97.5% after printing, while, for Bioinks E and F, this value was calculated at 93%. In the extreme laser fluence of 900 mJ/cm^2^, an average survival of 88% (from the examined bioinks) was calculated.

### 3.4. Influence of Laser Fluence on the Printed Volume and Droplet Size

It is known that the key parameter in controlling the LIFT process is the laser fluence [55,56]. The effect of the cell concentration on the printed droplet size was investigated at different laser fluences. The laser fluences ranged from the minimum needed for laser printing, to very high laser fluences causing the strong splashing of the droplets. The estimation of the printed volume was based on two types of experiments: single-droplet printing and multiple-droplet printing, in line configuration. As demonstrated in Figure 2 and Figure 3, the different cell-free/laden bioinks show ideal jet-impingement printing when a single breakup occurs, under a distance between the donor–receiver substrate of 500 μm. The droplet diameter increases almost linearly as the laser fluence increases.

**Figure 8:**
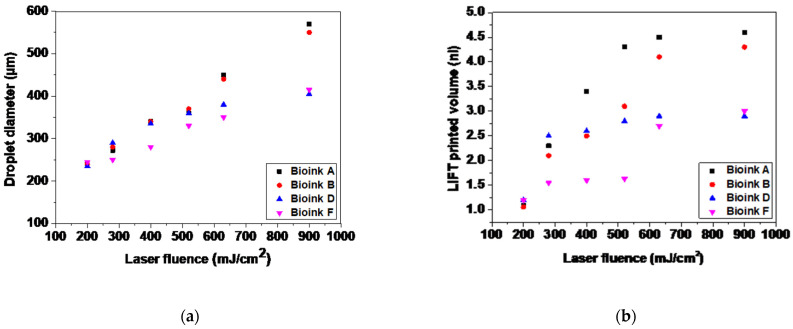
(**a**) LIFT-printed droplet diameters as a function of laser fluence; (**b**) LIFT-printed volume as a function of laser fluence.

In Figure 8, the relationship between the laser fluence and the droplet diameter is depicted. The droplet diameter increases as the laser fluence increases. In addition, we observed that, given a fixed fluence, the droplet diameter changes with the cell concentration. In the case of the cell-free bioinks, the droplet diameter increased from ~240 μm at 200 mJ/cm^2^, to ~560 μm at 900 mJ/cm^2^. On the other hand, at a given cell concentration, the transferred droplet was linearly dependent on the laser fluence, and the slope of the linear relationship between the transferred droplet and the laser fluence was dependent on the cell concentration. The use of cell-laden bioinks resulted in droplet diameters that increased from ~240 μm at 200 mJ/cm^2^, to ~400 μm at 900 mJ/cm^2^. The lower concentration bioinks resulted in larger printed droplet diameters. On the basis of our experimental measurements, viscosity is also an important factor. The comparable droplet diameters, as a function of the laser fluence for the cell-free bioinks, are explained by their close measured viscosity values (e.g., 2.17 mPa.s for Bioink A, and 2.37 mPa.s for Bioink B). The lower viscosity bioinks resulted in larger printed droplet diameters at constant experimental parameters [45]. Because we do not have experimental measurements for the viscosity of the cell-laden bioinks, our hypothesis is that the printed droplet diameter could be affected by the cell concentration in these bioinks.

In Figure 8b, we can observe the progression of the droplet volume and its nearly linear relationship with the laser fluence. At this point, it is important to emphasize that it is possible to laser print well-defined droplets for a wide range of cell concentrations (and cell-free solutions), something difficult to achieve using other bioprinting techniques, such as inkjet printing.

### 3.5. Influence of Distance between Donor and Receiver Substrate on the Jet Development and The Morphology of the Printed Droplets

It has been recognized that the distance between the donor–receiver substrate plays an important role in determining the printing quality during LIFT printing. The time-resolved images of Bioink F laser printing under constant laser fluence were further analyzed to assess the effect of the distance between the donor–receiver substrates.

**Figure 9:**
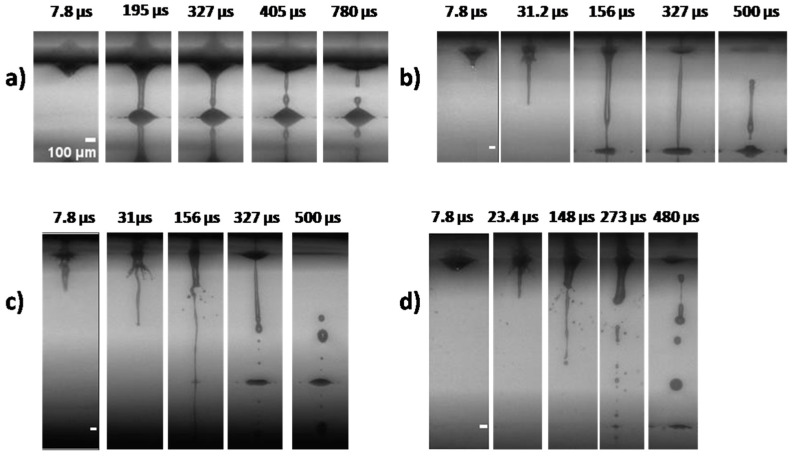
Time-resolved images of the LIFT Bioink F as a function of distance between the donor–receiver substrate: (**a**) 0.5 mm; (**b**) 1.0 mm; (**c**) 1.5 mm; and (**d**) 2.0 mm.Scale bar: 100 μm.

As demonstrated in Figure 9, the printing condition may vary from jet-impingement printing with a single breakup, to jet-impingement printing with multiple breakups, and even to droplet-impingement printing, when the distance between the donor–receiver substrates increases. In particular, for Bioink F at 500 mJ/cm^2^, the impingement type can be jet-impingement printing with a single breakup (for a 500-μm distance), jet-impingement printing with multiple breakups (for a 1000-μm distance), and droplet-impingement printing (for 1500- and 2000-μm distances). These results suggest that, for distances between the donor–receiver substrate smaller than the jet breakup length, the jet can reach the receiver substrate only with a single droplet without fragmentations, which may lead to the optimal printing quality (Figure 9a,b), whereas, for longer distances, the multiple fragments resulting from the jet breakup are transferred and secondary droplets are dispersed over the primary printed droplet on top of the receiver substrate because of the splashing (Figure 9c,d). Generally, a minimum distance between the donor–receiver substrates is recommended for specific applications to print a single droplet, e.g., to transfer a single cell [57]. Nevertheless, it should be noted that the optimal distance may not be easily determined during LIFT printing. If the receiver substrate is placed too close to the material on the donor substrate, the printed material on top of the receiver substrate may get in touch with the liquid material, which is on the tip of the donor substrate because of surface tension. Under such conditions, a greater distance is required to ensure the complete separation between the material under transfer and the deposited material, even if that means the less preferable condition of laser printing. It is further noticed that, if the distance of the substrates is small enough, there may not be enough space for the laser –induced bubble to produce a jet, resulting in an unsuccessful deposition [58].

**Figure 10:**
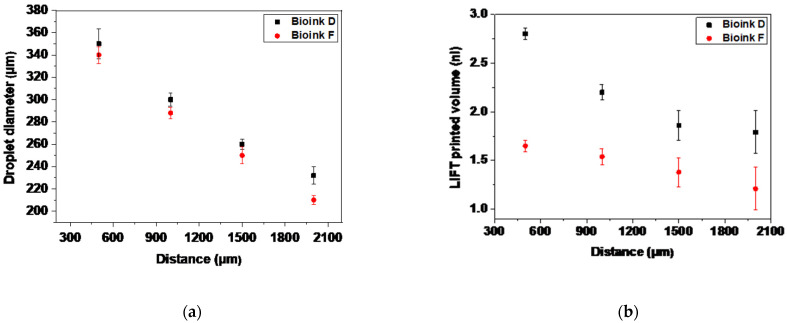
(**a**) LIFT-printed droplet diameter; and (**b**) LIFT-printed volume as a function of distance, at 500 mJ/cm^2^. Error bars depict STDEV, *n* = 4 droplets per condition.

The effects of different distances on the laser printing process, in terms of the printed droplet size and the volume, were studied during the LIFT printing of Bioinks D and F at 500 mJ/cm^2^. As shown in Figure 10a, there is a difference in the droplet diameter when the distance between the donor–receiver substrate is varied from 500 μm to 2000 μm. As observed, the droplet size decreases ~35% when the distance increases from 500 μm to 2000 μm. Similarly, the printed volume decreases when the distance increases (Figure 10b). In particular, when the distance increases over 1500 μm, the printing regime has converted from jet- to droplet- impingement printing. As a result, the formation of dispersed secondary droplets over the receiver substrate reduces the final droplet in terms of the volume and morphological characteristics. However, previous studies on the LIFT printing of hydrogels, in terms of the printed droplet size and volume as a function of the distance between the donor–receiver substrate, have shown that there were no significant changes in droplet sizes or volumes [43].

## 4. Conclusions

The LIFT printing of different cell-laden bioinks (and cell-free bioinks as models) has been investigated as a function of three key parameters: cell concentration, laser fluence, and the distance between the donor–receiver substrate using a time-resolved imaging technique of printed droplets. The printing regimes during laser printing are classified into the subthreshold, well-defined printing (with or without bulgy shape), and plume regimes. The well-defined printing conditions are identified as: droplet-impingement, jet-impingement with multiple breakups, and jet-impingement printing with a single droplet. In general, it was demonstrated that, for better printing quality, it is preferable to have jet-impingement printing with a single breakup; however, when the distance increases, the printing category may change from the ideal jet-impingement printing with a single breakup to jet-impingement printing with multiple breakups and the formation of secondary droplets. Furthermore, it was observed that, as the cell concentration of the bioink increases, the droplet size and velocity decrease, compared to the relevant cell-free bioinks. Finally, under appropriate conditions, high-cell-concentrated bioinks can be printed with very high cell survival.

Future studies are of great interest in understanding the droplet formation process during LIFT printing, such as: (i) How to simulate the hydrodynamic jet formation process and its parameters, such as the velocity, ligament length, breakup time, and droplet size during the LIFT of low-viscous materials; and (ii) To compare the jetting dynamics during the printing of low cell-laden and cell-laden viscoelastic bioinks with experimental observations.

## Figures and Tables

**Table 1:** Composition of the bioinks.

Bioink	Composition	Viscosity (mPa.s)	Density (g/cm^3^)
A	Dulbecco’s Modified Eagle’s Medium (DMEM)	2.1755	1.0092
B	Dulbecco’s Modified Eagle’s Medium (DMEM) (+10% glycerol)	2.3767	1.0360
C	DMEM + MDA-MB-468 (2.5 × 10^4^ cells/μL)	-	-
D	DMEM (+10% glycerol) + MDA-MB-468 (2.5 × 10^4^ cells/μL + 10% glycerol)	-	-
E	DMEM + MDA-MB-468 (7.5 × 10^4^ cells/μL)	-	-
F	DMEM (+10% glycerol) + MDA-MB-468 (7.5 × 10^4^ cells/μL + 10% glycerol)	-	-
G	DMEM + MDA-MB-468 (20 × 10^4^ cells/μL)	-	-

**Table 2:** Cell survival after transfer of different bioinks at 500 mJ/cm^2^.

Bioink	Printed Cells	Dead Cells
C	121 ± 9	7 ± 2
D	110 ± 4	11 ± 5
E	180 ± 16	9 ± 5
F	185 ± 14	12 ± 7

**Table 3:** Cell survival after transfer of different bioinks at different laser fluences.

Laser Fluence (mJ/cm^2^)	Bioink	Printed Cells	Dead Cells
400	Bioink C Bioink D Bioink E Bioink F	65 ± 5 90 ± 6 163 ± 10 160 ± 12	0 4 ± 3 7 ± 6 15 ± 9
900	Bioink C Bioink D Bioink E Bioink F	83 ± 7 104 ± 10 240 ± 14 260 ± 20	12 ± 5 8 ± 6 35 ± 6 25 ± 9

## Data Availability

Data is contained within this article.
